# Unfolding rotational tectonics and topographic evolution from localized verses diffuse plate boundary counterparts

**DOI:** 10.1038/s41598-024-58921-y

**Published:** 2024-04-08

**Authors:** Bhaskar Kundu, Frank Zwaan, Batakrushna Senapati

**Affiliations:** 1 Department of Earth and Atmospheric Sciences, NIT Rourkela, Rourkela, 769008 India; 2 Institute of Geological Sciences, University of Bern, Baltzerstrasse 1+3, 3012 Bern, Switzerland; 3 Helmholtz Centre Potsdam - GFZ German Research Centre for Geosciences, Telegrafenberg, 14473 Potsdam, Germany; 4 Department of Geosciences, University of Fribourg, Ch. du Musée 6, 1700 Fribourg, Switzerland

**Keywords:** Central Indian Tectonic Zone, Gakkel Ridge, Chersky Range, Analogue modelling, Localized rifting, Diffuse deformation, Euler pole, Tectonics, Solid Earth sciences, Geodynamics, Planetary science, Structural geology

## Abstract

We present a kinematic model developed from geodetic observations, topography analysis and analogue tectonic modelling results, which reveals a striking similarity between the rotational tectonic settings of the Gakkel Ridge-Chersky Range system in the Arctic, and the Central Indian Tectonic Zone within the Indian subcontinent. A crucial aspect of large-scale extensional rift systems is the gradual variation of extension along the rift axis, due to plate rotation about a Euler pole, which may lead to contraction on the opposite side of the Euler pole to form a rotational tectonic system. Our geodetic and topographic analysis, combined with the reanalysis of analogue tectonic modelling results demonstrates such rotational tectonic plate motion in both the Arctic and Indian case. However, the plate boundary between the North American and Eurasian Plates as represented by the Arctic Gakkel Ridge-Chersky Range system is strongly localized, whereas the Central Indian Tectonic Zone that separates the North and South India Plates involves diffuse deformation instead. Furthermore, in both the Arctic and Central Indian we find that the relative Euler rotation pole is located near an indenter-like feature, which possibly controls the present-day rotational tectonics and contrasting topography on opposite sides of the Euler pole.

## Introduction

**Figure 1:**
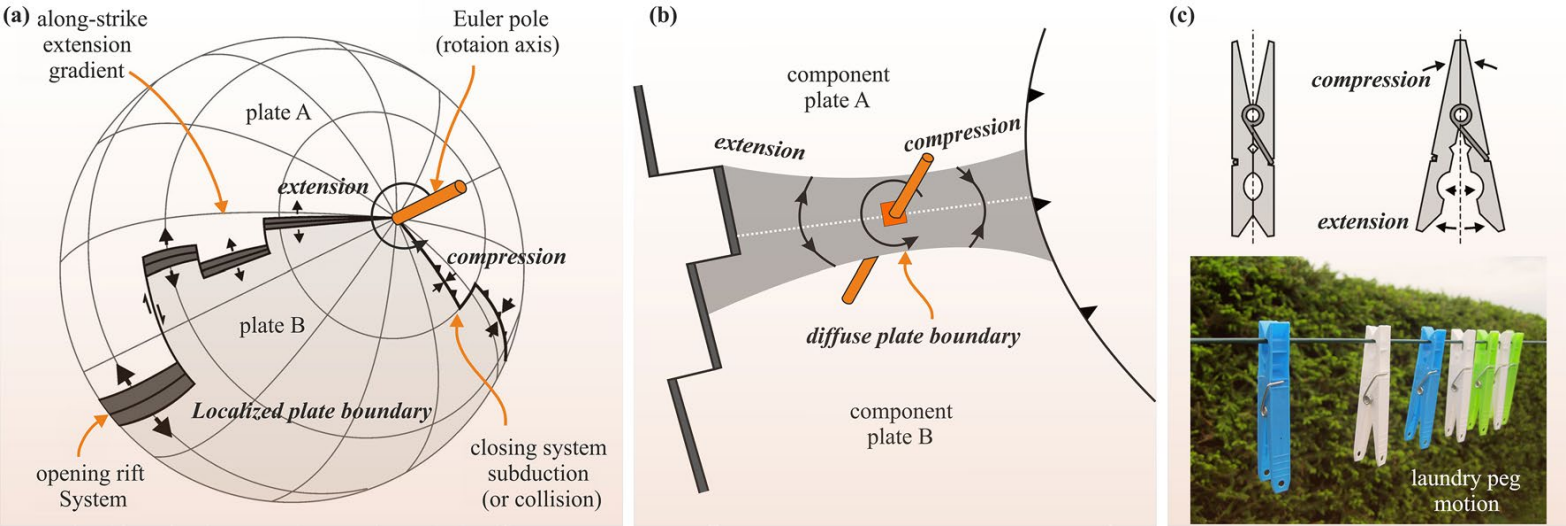
Basics of rotational tectonic settings. (**a**) Rotational tectonics on a global scale. The pivoting motion of the rift system is characterized by a Euler (rotation) pole. (**b**) Schematic representation of a composite plate consisting of two component plates (A and B) separated by a diffuse plate boundary. (**c**) Rotational tectonics can be described by the opening and closing on opposite sides of the rotation axis or relative Euler pole, similar to laundry peg motion^3^. This figure was generated using Corel Draw graphical application (version 22.2.0.532 URL: https://www.coreldraw.com/en).

A characteristic aspect of rifting in continental or oceanic settings is the variation of deformation patterns along the rift axis during rift propagation^1–3^ (Fig. 1). Rifts often nucleate along lithospheric weaknesses and eventually propagate away from these weaknesses, into the stronger parts of the lithosphere, where past extension was accommodated by distributed deformation^4^. This propagation is linked to varying extension rates along the rift axis as predicted by rotational tectonics about a Euler Pole^3^ (Fig. 1a,b). As a consequence of such rotational motion, extensional and contractional structures may develop on the opposite sides of the rotation axis or Euler pole. Some well-known examples of rift systems developing in rotational tectonic settings, often with contractional features on the opposite side of the rotation axis, are the Taupo Rift in New Zealand^5^, the Woodlark Basin of Papua New Guinea^6,7^ and the Gakkel Ridge in the Arctic^3,8^.

**Figure 2:**
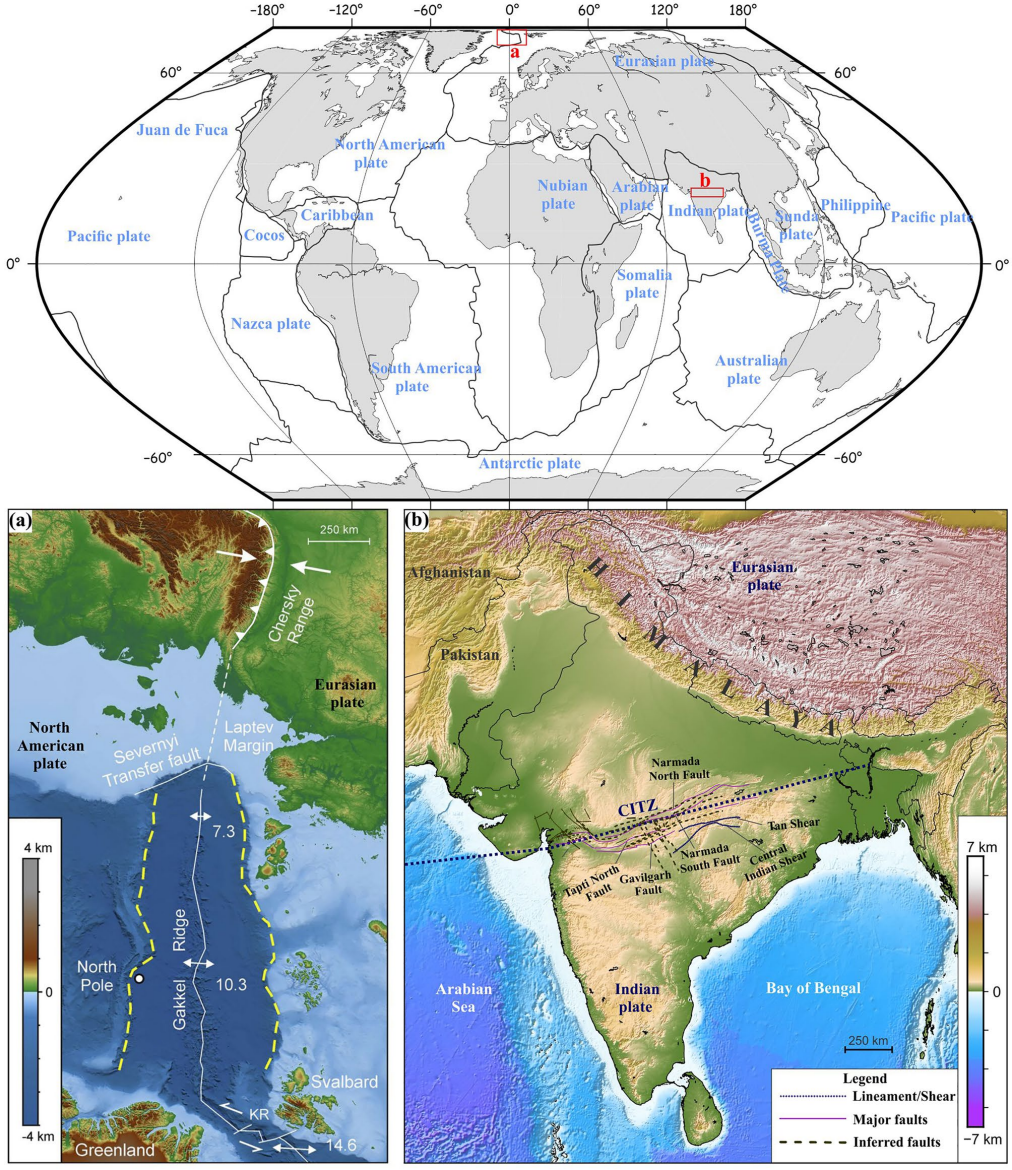
(**a**) World map showing the locations of Gakkel Ridge-Chersky Range system, and the Indian subcontinent study area. (**b**) Bathymetric and topographic map of Arctic plate boundary system showing an extension rate gradient along the Gakkel Ridge (in mm/year, after Dick et al.^12^) and compression along the Siberian Chersky Range (after Imaeva et al.^16^. The white line indicates the rift axis or plate boundary between the North American Plate and the Eurasian Plate. The dotted yellow line indicates the border of the extensional system. KR: Knipovitch Ridge. (**c**) General tectonic features of Central India showing the different fault segments and lineament along Central Indian Tectonic Zone (CITZ)^23,67^. This figure was generated using Generic Mapping Tools (version 5.2.1; URL: http://gmt.soest.hawaii.edu/).

In this article, we present new analyses to support the recently proposed interpretation of present-day crustal deformation of the Central Indian Tectonic Zone (CITZ) as a new type of rotational tectonic system involving a diffuse plate boundary between the North India Plate and South India Plates^9^, and compare it to the Gakkel Ridge-Chersky Range rotational tectonic system in the Arctic (Fig. 2). Our endorsement of this new rotational tectonic interpretation, rather than the previous general assumption that the Indian Plate behaves as a single rigid block (REF)^10^, of the CITZ is based on a multidisciplinary approach, in which we first analyze plate motions using Global Navigation Satellite System (GNSS) velocities from the Arctic region (as a classic rotational tectonic system), and compare these with a geodetic analysis of the CITZ^9^, Moreover, a new kinematic model is developed by re-estimating Euler poles and angular velocities. We furthermore perform a topography analysis for the both cases (i.e., CITZ and Gakkel Ridge in the Arctic) and reanalyse the analogue model of rotational tectonics proposed by Zwaan et al.^3^. The outcomes of this approach validate the new interpretation of the CITZ as a currently active rotational tectonic system in Central India. We furthermore propose that this rotation between the North Indian and South Indian Plates may be caused by an indenter-like feature stuck below the diffuse plate boundary, which acts as a pivot point, similar to the impact of an indenter-like feature in the Arctic.

## Tectonic setting

### Gakkel Ridge-Chersky Range system in the Arctic

The Gakkel Ridge or Arctic mid-oceanic ridge represents a seismo-tectonically active divergent plate boundary zone between the North American Plate and Eurasian Plate, stretching along the length of the Eurasian Basin^3,11,12^ (Fig. 2a). The ridge was named after the Soviet researcher of the polar region, Yakov Levich Gakkel, who confirmed its existence and approximated the geographical location of the ridge during a Soviet Artic expedition in ~ 1950. In the Laptev Sea, where the Gakkel Ridge enters the continental lithosphere of the Eurasian Plate, the localized deformation of the ridge is diffused into various rift basins^2,13^. The Gakkel Ridge represents a gradient of slow spreading rates along its ~ 1800 km long axis as present-day extensional rate decreases from ~ 12 mm/year near Svalbard to ~ 7 mm/year near the Laptev Margin, offshore Siberia^12,14–16^ and the ridge system represents an active source of earthquakes^17^. Whereas rifting along the Gakkel Ridge is due to divergent plate movement between the North American Plate and Eurasian Plate, contraction occurs on the other side of the Euler pole to form the Chersky Range^16^.

The tectonic history of the Gakkel Ridge is interpreted from linear magnetic anomaly data within the Eurasian oceanic basin^18–20^. Continental breakup initiated in the Eocene (~ 55–33 Ma) due to sea-floor spreading at a maximum rate of 20 mm/yearr. Afterwards, northward movement of the Lomonosov Ridge initiated with respect to the Barents-kara shelf, when the Siberian end of the Lomonosov Ridge moved eastward with respect to the Laptev Sea region, along a sheared margin^21^. Due to abrupt change in global plate tectonics ~ 33 Ma, a period of significant decrease in plate movements occurred, and the rate of spreading between the North American Plate and Eurasian Plate dropped to ~ 5 mm/year^22^. In Middle-Late Miocene to Middle Pleistocene times plate divergence accelerated in the Eurasian Basin, after which the present-day plate boundary arrangement was achieved. During this reactivation of rifting in the Laptev Sea, the formation of the Moma Rift in the Chersky Range area was initiated and furthermore, divergence have been active at a low rate in the vicinity of the relative Euler pole that is situated near the tip of the rift^12,22,23^.

### Central Indian Tectonic Zone

The CITZ is a significant seismo-tectonical active zone within the Indian plate’s interior region, which stretches ENE–WSW for more than 1000 km, is ~ 400 km in width and dissects the Indian Peninsula into a northern and a southern crustal block^24,25^ (Fig. 2b) that can be identified as a diffuse deformation zone^9^. The precursor of the CITZ was possibly formed by a Precambrian continental–continental collision phase within Peninsular India and this zone has witnessed various major events such as the extrusion of the voluminous Deccan flood basalts in the late Cretaceous^25–27^. Nowadays, this major tectonic features splits the whole Indian peninsula into two parts, where the northern part covers the Bundelkhand Craton of Archean age and the Aravalli Craton of Proterozoic age, whereas the southern part includes the Dharwar Craton (~ 3.6–2.5 Ga), Bastar Craton (~ 3.5 Ga) and Singhbhum Craton (~ 3.3–3.2 Ga)^24^. Several major deep-seated ENE–WSW trending faults are found along the Narmada-Son paleo rift zone in the western part of the CITZ, among which those of the Narmada rift and Tapti rift, located to the south of the CITZ^23^. The Narmada-Son paleo rift zone has been identified as a failed rift arm^28,29^ and it has been proposed that this structurally weak zone may have been reactivated by igneous activity at the Cretaceous-Tertiary boundary. Unusual moderate to high magnitude seismicity occurrences (M > 6) have been reported in this plate interior region of the Indian subcontinent^9^. Hence, the nature of present-day crustal deformation, earthquake generation processes and its relation with the associated topography build-up along this paleo rift zone within Central Indian remains poorly understood.

## Results

### Localized rotational tectonics in the Arctic

In order to characterize crustal deformation, rotational tectonic processes, and their relation with topography build-up along the Gakkel Ridge-Chersky Range in the Arctic, we have investigated geodetic dataset from the adjacent plate boundaries. We estimate the absolute as well as relative plate motion and Euler rotation parameters of the North American Plate and Eurasian Plate, considering a large number of geodetic data sets from previously published sources (i.e., ~ 684 stations for the Eurasian Plate and ~ 2223 stations for the North American Plate) (See the Supporting Documents and Tables S1, S2).

**Figure 3:**
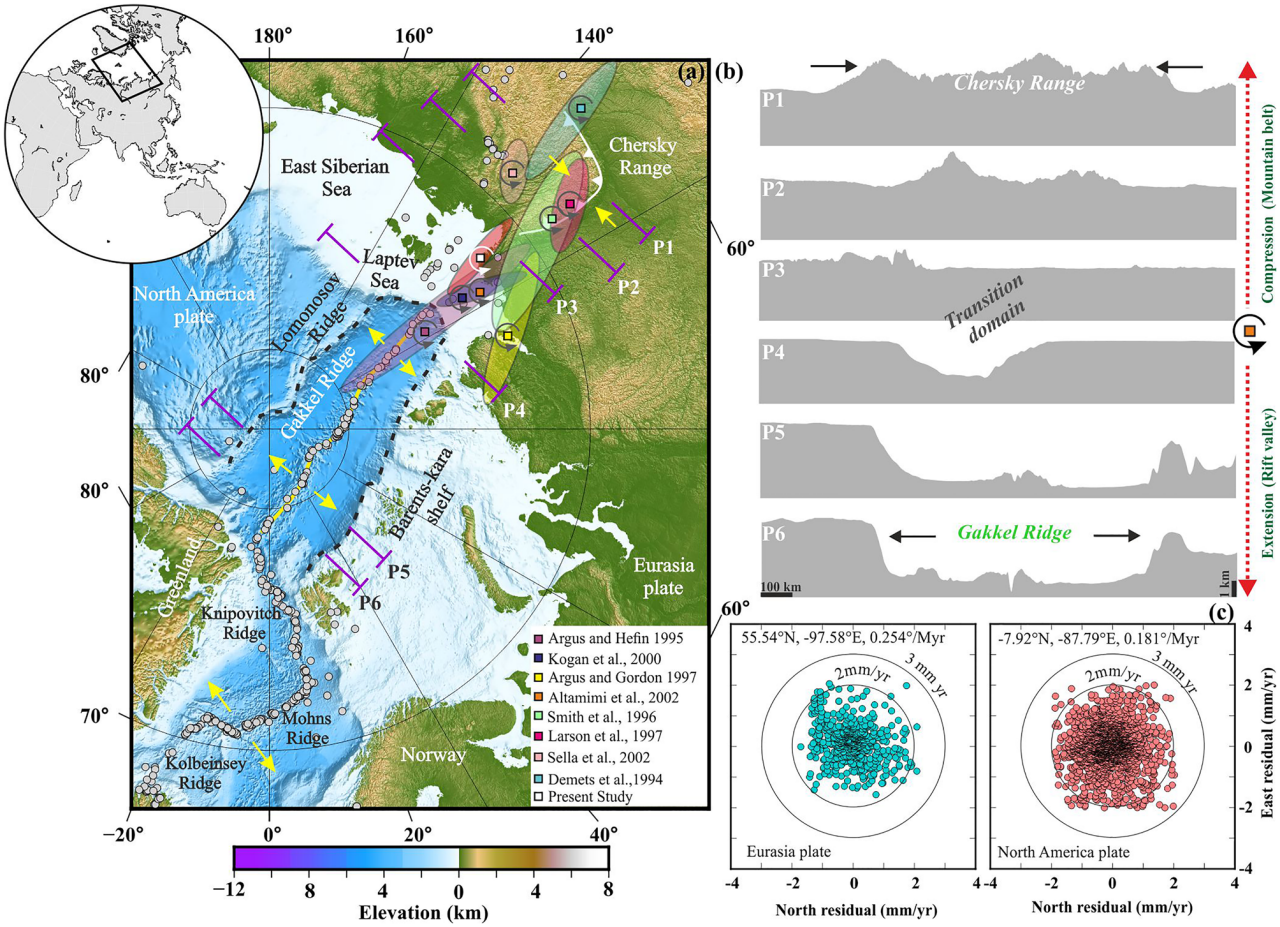
Results of the geodetic and topographic analysis of the Arctic region. (**a**) General tectonic setting in the Arctic. The topographic swath profiles (P1–P6) across the Gakkel Ridge-Chersky Range system are marked in magenta and are shown in panel (**b**). Eurasia-North America relative rotation poles are marked with different colours with pole error ellipse (95% confidence level) convention as defined by the respective authors (shown in inset), where the newly estimated relative pole is marked by a white rectangle. Yellow arrows indicate the extension and contractional deformation regimes of the Arctic rift system. Dotted black lines indicate the boarder of the Eurasian Basin. The epicentral distribution of earthquakes (M > 5) from USGS are marked in grey. Note that extension gradients along the Arctic Gakkel Ridge (indicated by longer yellow arrows towards Greenland, see also Fig. 1a) and contraction at the Siberian Chersky Range suggest a rotational motion about a North America-Eurasia rotation axis or relative pole. (**b**) Topographic swath profiles (i.e., P1–P6 marked in panel (**a**)) across the Gakkel Ridge-Chersky Range system, which marks the Eurasia-North America localized plate boundary zone. (**c**) Residual plate motion and absolute Euler pole parameters of Eurasian and North American Plate, respectively. Turquoise and light red colour symbols represent the cGPS sites, which are considered in the estimation of the Euler poles for both plates. This figure was generated using Generic Mapping Tools (version 5.2.1; URL: http://gmt.soest.hawaii.edu/) and Grapher graphical application (version 16.6.478 URL: https://www.goldensoftware.com/products/grapher).

From these geodetic constraints, we observe that the present-day absolute Euler pole of the North American Plate is located at − 7.92 ± 0.115° N, − 87.79 ± 0.030° E, with an angular rotation rate of 0.181 ± 0.0003°/Myr. Similarly, the Euler rotation pole of Eurasia is located at 55.54 ± 0.103° N, − 97.58 ± 0.138° E, with an angular rotation rate of 0.254 ± 0.0004°/Myr. The residual velocity plots of the North American and Eurasian Plates indicate that internal deformation within both plates is very minor (~ 2–3 mm/year) (Fig. 3c). Also, in order to understand the motion accommodated along the Gakkel Ridge to Chersky Range, the relative Euler pole (i.e., in between North American Plate and Eurasian Plate) (Fig. 3a) is estimated to be situated at 75.40 ± 1.7° N, 124.2 ± 1.5° E, with a rotation rate of 0.233 ± 0.008°/Myr, which is consistent with the results from earlier investigations (Table S3). The locations of all the previously estimated relative Euler poles, along with our new estimation (Fig. 3a), indicate a rotational tectonic system with contrasting deformation on either side of the Euler pole. This is in excellent agreement with the observation that moving towards the Euler pole, the extensional deformation of the Gakkel Ridge is gradually decreased, and is exchanged by contractional tectonics along the Chersky Range on the other side of the Euler pole. This difference in tectonic setting on opposite sides of the Euler pole in this rotational tectonic system is excellently illustrated by topographic swath profiles (P1–P6 shown in Fig. 3a,b) across the Gakkel Ridge and Chersky Range, which clearly show the rift basins resulting from extension along the Gakkel Ridge, and the mountainous topography of the Chersky range as a result of the contractional tectonics there^15^.

### Diffuse rotational tectonics in the Central Indian Tectonic Zone (CITZ)

**Figure 4:**
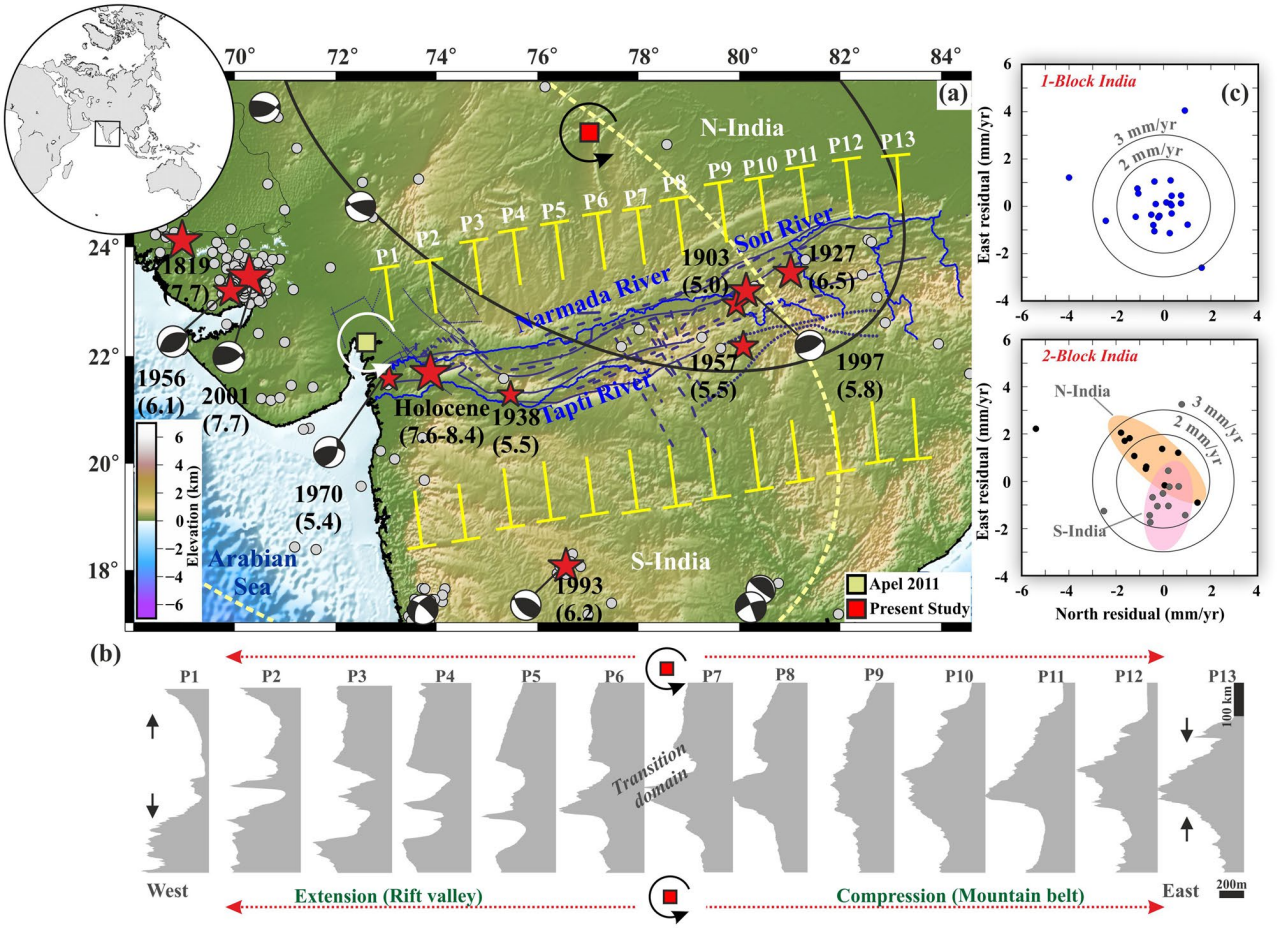
Results of the geodetic and topographic analysis of Central India. (**a**) General tectonic map of Central Indian Tectonic Zone. The topographic profiles (i.e., P1–P13) across Central India are marked in yellow. Faults are marked by deep blue colour (after Joshi et al.^68^; Deshpande and Gupta^24^), and major rivers (Narmada, Son, Tapi) are marked in blue color. The earthquakes from USGS are shown in grey. Significant and historical earthquakes are shown by red colour stars with their available focal mechanisms. (**b**) Swath topographic profiles (within 73°–84° E longitudinal range) across the Central Indian Tectonic Zone at 50 km intervals (i.e., P1–P13 marked in **a**). Note the topography contrast between either side of the relative pole. (**c**) Residual plate motion by considering a 1-Block (Considered GPS stations marked as blue) and a 2-Block (Considered GPS stations marked as black for N-India and grey for S-India) Indian plate. This figure was generated using Generic Mapping Tools (version 5.2.1; URL: http://gmt.soest.hawaii.edu/) and Grapher graphical application (version 16.6.478 URL: https://www.goldensoftware.com/products/grapher).

Next to the localized rotational tectonics situation in the Arctic, we present an overview of our results from the CITZ that represent a zone of diffuse deformation. Our geodetic constraints include 28 continuous GPS velocities from stations within the continental Indian plate interior that are used to estimate the rotation parameters of the Indian Plate (see Supplementary Information, Table S4). We first estimate the location of the Euler pole of the Indian Plate as a whole, which is found to be situated at 50.82 ± 0.2° N, 9.28 ± 1.47° E with an angular rotation rate of 0.538 ± 0.004°/Myr in a ITRF2014 reference frame. Moreover, from the residual velocity plot presented in Fig. 4c (1-Block Indian panel), the internal Indian Plate deformation is ~ 1–2 mm/year, indicating that the plate possibly behaves in a rigid block manner.

Although the above geodetic analysis seems to suggested that the Indian plate is behaving in a rigid block manner, the occurrence of unusual strong earthquakes of M > 6.0 inside the continental parts of Indian plate implies that the entire plate is in fact not behaving as a single rigid block. Hence, we also test the ‘composite’ plate hypothesis put forward by Sen et al.^9^ which involves two ‘component’ plates (i.e., N-India and S-India) separated by the Narmada-Son paleo rift zone.

Therefore, we re-estimate the location of the Euler pole defining potential independent plate motions north and south of the CITZ. Our analysis shows that there is a significant difference in longitudinal position (~ 10° apart) of Euler poles between the two inferred parts of the Indian plate (the North India and the South India Plates), although overall latitudinal position and rotation rate remains same as presented for the single Indian Plate (Tables S4, S5, Fig. 4c). This indicates that the South India Plate is moving relatively faster than the North India Plate, with an overall N–S shortening. Residual velocity plot of composite Indian plate (2-Block India panel in Fig. 4c) indicates two distinct clusters of segregation for the North India Plate and South India Plate, and overall internal deformation of the Indian plate is significantly lower (~ 3 mm/year). Considering our ‘composite’ Indian plate hypothesis, we have computed relative pole of rotation for motion for the North India Plate and South India Plate, that lies very close to the boundary between both plates (25.92 ± 6.8° N, 77.09 ± 4.0° E) with an angular rotation velocity that is ~ 90% slower than that of the Indian plate (Fig. 4a).

Furthermore, this relative pole of rotation between these two component plates predicts contrasting deformation styles on both sides of the rotation pole along Narmada-Son paleo rift, with shortening in the east (~ 0.1–1.2 mm/year) and extension in the west (~ 1.2–1.9 mm/year) (Fig. 4b). This contrasting nature of present-day deformation inferred from our analysis, appears to be in good agreement with the contrasting topography along the plate boundary (i.e., mountains and rift valley on the eastern and western side respectively), illustrated by swath topographic profiles (Fig. 4b). We also project the predicted velocity by considering a Single Indian Plate pole and two Indian Plate poles (i.e., North-Indian and South-Indian plate pole) and notice that in the case of two Indian Plate poles, the South-Indian Plate is moving faster than the North-Indian Plate. We also observe that there is a velocity change along Narmada-Son diffuse plate boundary (Fig. S1). However, we find no such change in velocity in the case of a Single Block India pole (Fig. S1). This also supports our composite Indian plate hypothesis.

Moreover, to ensure more statistical robustness of the present kinematic model, we have calculated the Root Mean Square Error (RMSE) between GNSS velocities and kinematic velocities for the present kinematic model and previously proposed kinematic model (for detail see material and method section, Tables S6–S8). We have noticed that RMSE values between GNSS velocities and kinematic velocities for the present kinematic model is lower than the previously proposed kinematic model (Tables S6–S8). Hence we have suggested that the present kinematic model has better fits the GNSS velocities.

We thus find very similar rotational tectonic features in both the Arctic as in Central India, and we can infer that the Central Indian Tectonic Zone is a diffuse plate boundary counterpart to the localized Gakkel Ridge-Chersky Range system.

### Topography development and associated rotational tectonics: insight from 4D analogue tectonic model analysis

**Figure 5:**
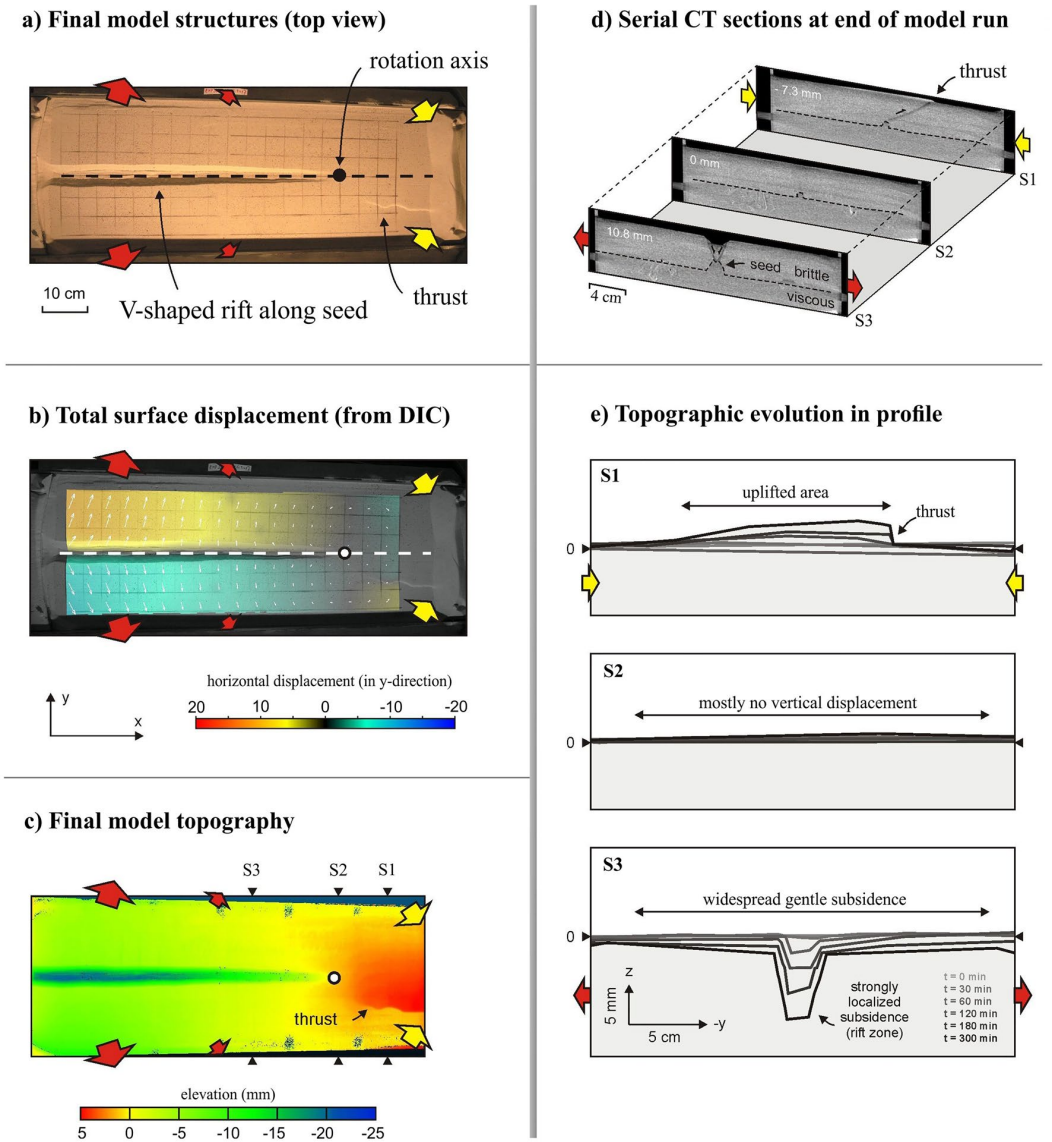
Results from an analogue modelling of a rotational tectonic system. (**a**) Final surface structures (top view) developed along a linear seed representing a structural weakness (seed trace is indicated by dotted line). The rotation axis is marked by a black circle. Red and yellow arrows indicate the extension and contraction on the opposite sides of the rotation axis. (**b**) Digital Image Correlation (DIC) analysis of total cumulative horizontal surface displacements (marked by white arrows). A dotted line indicates the seed trace. The rotation axis is marked by a white circle. (**c**) Final topography model (rotational extension) indicating the vertical displacements along the rift axis at the final stage. (**d**) Serial CT sections (S1, S2, and S3 are the different cross sections, the location is shown in **c**) at the end of model run reveals contrasting topographic architecture on the opposite sides of the rotation axis. (**e**) Final 3D topographic profiles (S1, S2, and S3) and vertical motion analysis along the rift axis show subsidence (extension) and uplifted (contraction) region along with vertical CT sections. Locations of sections S1, S2 and S3 are shown in (**c**). This figure was generated using Generic Mapping Tools (version 5.2.1; URL: http://gmt.soest.hawaii.edu/).

We revisit a brittle-viscous experimental model of rotational tectonics completed by Zwaan et al.^3^, to get better insights into the topographic evolution of a rotational tectonic systems (Fig. 5). The set-up used by Zwaan et al.^3^ consisted of a foam base stacked between longitudinal sidewalls on top of a fixed table that allowed the foam to move freely at the base. The longitudinal sidewalls could move by means of precise computer-controlled motor, and were linked to a rotation pole below the model. As a result, outward motion of the sidewalls on one side of the rotation pole resulted in extension of the foam base there, as well as inward sidewall motion and contraction of the foam base on the other side of the pole. The rate of this deformation increased away from the location of the rotation pole, thus reproducing a rotational tectonic system. This deformation was subsequently transferred into the brittle-viscous model layering overlying the foam base. Here a layer of quartz sand simulates the brittle upper continental crust, whereas a layer of viscous mixture represents the lower continental crust (Table S9). In order to localize the deformation along the centre of the model domain a linear ‘seed’ was used, which acted as a linear weak zone in nature. Zwaan et al.^3^ analyzed their models via time lapse top view photographs, which provided a qualitative visual analysis of surface deformation, but also allowed a quantitative analysis of surface displacement through digital image correlation (DIC) analysis^30,31^ (Fig. 5a,b). Furthermore, Zwaan et al.^3^ applied an X-ray Computed Tomography (XRCT) scanner to monitor 3D internal model evolution (Fig. 5d). XRCT data were also used to create topography maps of the model surface (Fig. 5c). Here, we reanalyze the XRCT imagery from Zwaan et al.^3^ in profile view, in order to monitor the topography evolution of the different domains of the model (i.e., in the extensional domain, at the rotation pole, and in the contraction domain). The results are subsequently compared to the topography profiles from the Gakkel Ridge-Chersky Range system in the Arctic, and the CITZ within the Indian Plate respectively.

The relation between rotational tectonics and topography evolution related to the analogue tectonic model results, complementing with our geodetic measurements, is presented in Fig. 5. The final model top view photographs depict the formation of an extensional regime (i.e., V-shaped rift basin) and compressional regime (i.e., mountain belt) on opposite sides of the rotation axis. Furthermore, the DIC results clearly indicates the horizontal displacement associated with rotational tectonics with increasing displacement away from the rotation axis (Fig. 5b). The final topography map illustrates the propagation of the localized rift basin toward the rotation axis, a general subsidence in the extensional regime and regional uplift as well as formation of a thrust on the other side of rotation axis in the compressional regime. Series of CT-scanned sections (e.g., S1, S2, and S3, locations marked in Fig. 5c) taken at the end of the model run reveal the variation in internal faulting and topography on either side of the rotation axis and overall structure varies along strike. Our new topography analysis along the three XRCT sections across the model axis (S1, S2, and S3) provides additional insights into the impact of rotational tectonics on topography (Fig. 5e). Widespread gentle subsidence as well as a strong localized subsidence occurs along the profile of S3, representing the extensional domain, however on the other hand, a wide uplift and the formation of a thrust faults are visible along the profile of S1, representing contractional domain. This spatial diversity in the topographic build-up and relation with rotational tectonics are in good agreement with our observations from the Arctic and Central India (Figs. 3, 4).

## Discussion

### Comparison between localized vs. diffuse plate boundary

Our new estimation of the relative pole in between North America and Eurasia shows consistency with earlier estimations (Table S3) and the relative Euler pole location and its angular rotation rate suggests an on-going rotational extension setting in the Arctic. As a result, the Gakkel Ridge meets the Laptev Margin, where localized extension changes to distributed deformation^2,3,13^ and finally joins with the Chersky Range, the other side of the Euler pole, while transforming into a compressional tectonic setting.

In the case of the Indian plate, several authors have suggested that the plate-interior deformation is also very limited ~ 1–2 mm/year, considering the plate to be behaving as a rigid block manner^29,31,32^. However, several studies^33–36^ report the occurrence of moderate to higher magnitude (M > 5) earthquakes (e.g., Holocene age Tapti earthquake, 1927 Son-valley, 1938 Satpura, 1970 Broach, 1997 Jabalpur earthquakes) within the central part of the Indian subcontinent, yet the mechanism of such unusual moderate to higher magnitude earthquakes within stable plate interior is elusive. In this study we suggest that, based on a multidisciplinary approach involving the integration of geographically well-distributed GPS datasets of longer data duration, topographic cross-sections across the diffuse deformation zone, the CITZ in Central India has segmented the Indian plate in two ‘component’ plates, i.e. that N-India and S-India Plate (Fig. S1).

Hence, the Gakkel Ridge-Chersky Range system in the Arctic and the CITZ in Central India are comparable with each other in several geodynamic aspects (Fig. 7). Firstly, both regions show rotational plate motion explaining the extensional and contractional deformation on opposite sides of the relative Euler pole. Secondly, both regions contain contrasting topography on either side of the relative rotation pole that are expected from deformation in rotation tectonic settings. However, an important difference between these two regions is that the CITZ is part of a diffuse plate boundary that involves a dispersed type of deformation, whereas deformation in the Artic concentrates along a narrow zone, revealing a localized plate boundary style.

### Possible cause of the rotation: a buoyant ridge or indenter?

**Figure 6:**
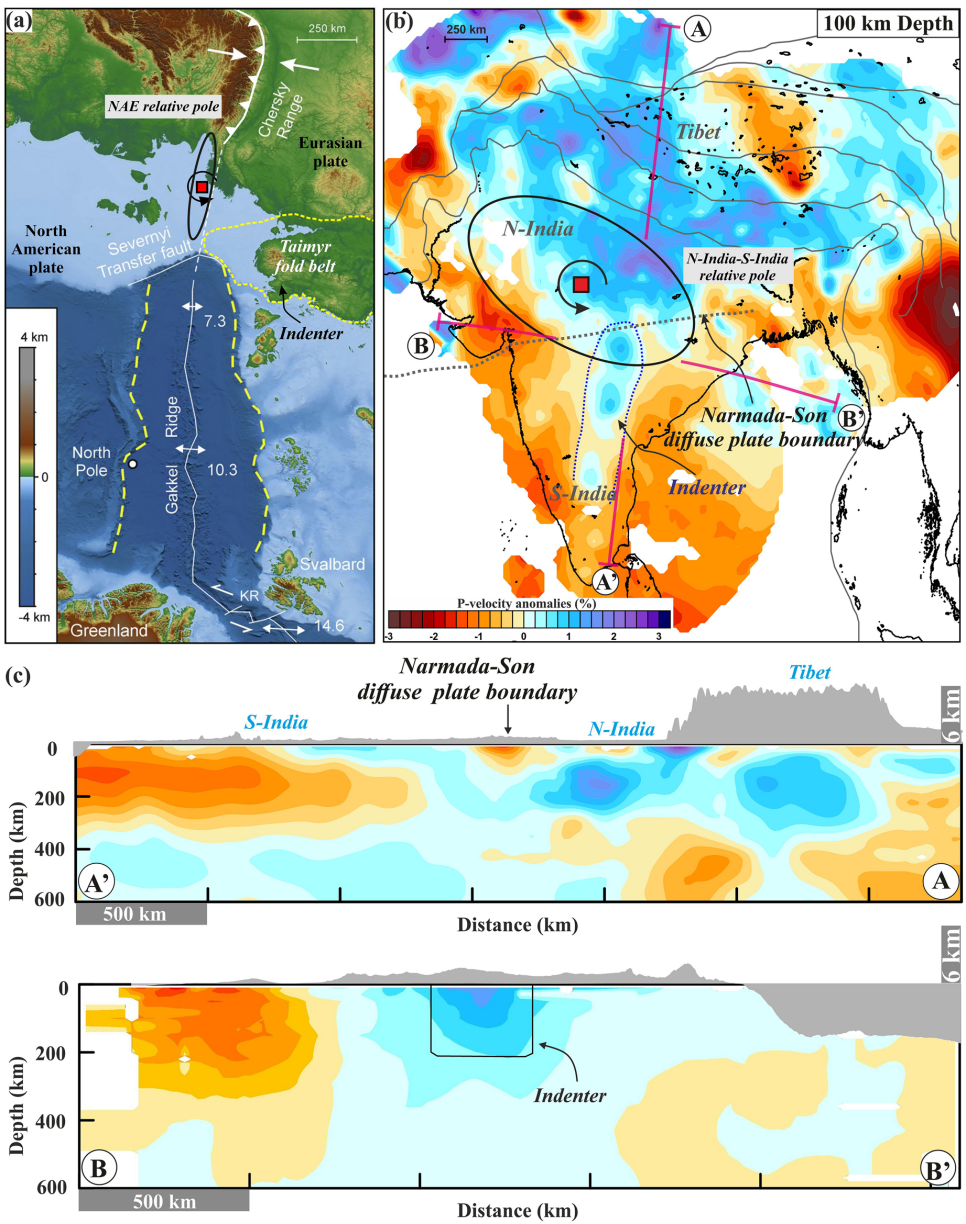
Possible indenter structures in the Arctic and Central India that serve as pivot points for rotational tectonic deformation. (**a**) Possible location of an indenter (i.e., Taimyr fold belt) adjacent to the presently estimated relative Euler pole of the North American Plate and Eurasian Plate (marked by small red square). (**b**) Possible location of an indenter in the Central Indian Tectonic Zone. P-wave velocity anomalies from the tomographic inversion model are observed at 100 km depth (modified from Koulakov et al.^69^). The vertical cross-sections (i.e., A–A′ and B–B′, marked in magenta) of the P-wave velocity anomalies are shown in (**c**). (**c**) Vertical cross-sections showing P-wave velocity anomalies at 100 km depth and topographic variation along the A–A′ and B–B′ profiles. Note that higher P-wave velocity anomalies indicate the location of the indenter/aseismic ridge (Marked in B–B′ profile) that possibly interacts with the Central Indian Tectonic Zone diffuse plate boundary, which is also very close to the location of the relative Euler pole. This figure was generated using Generic Mapping Tools (version 5.2.1; URL: http://gmt.soest.hawaii.edu/) and Surfer graphical application (version 13.6.618 URL: https://www.goldensoftware.com/products/surfer).

Aside from the geodetic constraints and analogue modelling results, we can use published data on lithospheric structure, and structural and seismic stratigraphic characteristics of the Gakkel Ridge-Chersky Range and the CITZ regions^3,23^, to interpret the possible cause of the rotational tectonics in these two regions (Fig. 6). Magnetic anomaly data along with geological cross section along the seismic profiles and associated seismic stratigraphic from the Artic^20,21^, reveal the presence of a structural indenter (i.e., Taimyr fold belt structural units) near the location of the relative Euler pole between the Eurasian and North American Plates (Fig. 6a). Similarly, seismic tomography data showing the lithospheric structure of the Indian plate clearly show that there are high P-wave velocity anomalies beneath the North India Plate, whereas below the South India Plate, the high Vp anomalies are less eminent and alternates with lower Vp anomaly (Fig. 6b). The P-wave velocity anomalies also suggest there is contrasting lithospheric structure (thicker and thinner) on either side of the CITZ. Moreover, there is evidence for the presence an indenter (or a NNE-SSW trending aseismic ridge) associated with the Southern Indian Plate (Fig. 6c).

**Figure 7:**
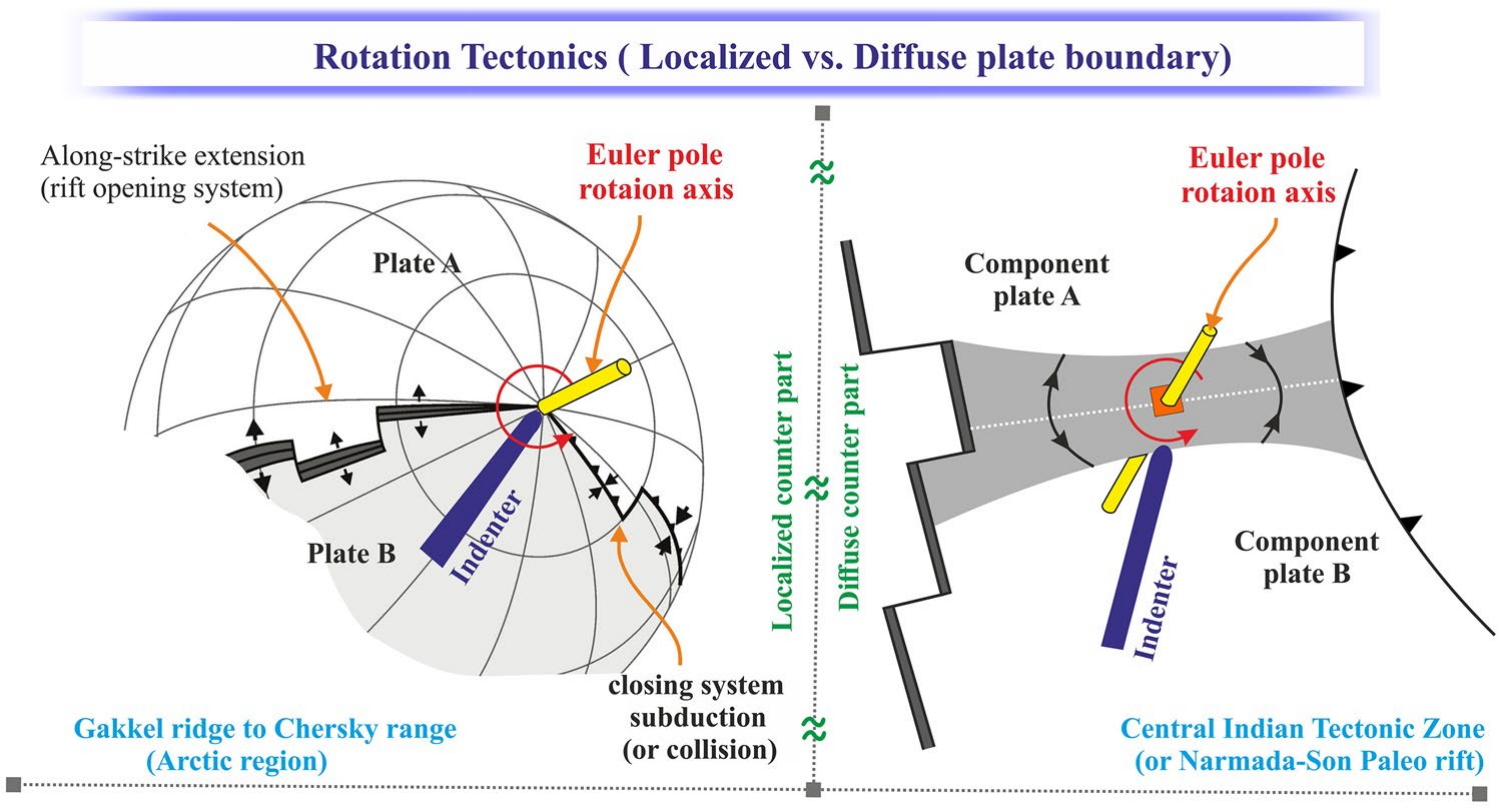
Models of indenter-controlled/affected rotational tectonics and contrast in deformation style (i.e., extension and compression on opposite sides of the rotation axis or relative Euler pole). The left and right panel shows the proposed rotational tectonics and deformation style of the Arctic region and central Indian tectonic zone, respectively, where the indenter controls the location of the Euler pole. This figure was generated using Corel Draw graphical application (version 22.2.0.532 URL: https://www.coreldraw.com/en).

The presence of such indenter-like structurers near the relative rotation pole in both the Arctic and the CITZ is likely not just a simple coincidence (Figs. 3, 4). In fact, we argue that such collision of the indenter (or the buoyant ridge) across this diffuse/localized boundary can acts a pinning point or pivot point by exerting a torque on the microplate (for diffuse plate boundary) or adjacent plate boundary zones (for localized plate boundary) (Fig. 7). We therefore suggest that the existence of a buoyant ridge in the Indian Plate and the structural indenter in the Arctic region are possibly the cause of the rotational tectonics observed in both regions. This interaction between the indenter and adjacent plate boundaries would explain several observations such as the relative pole location across two respective component plates, contrasting topography along the plate boundary (i.e. on opposite sides of the Euler pole), and the occurrence of frequent seismic activity of high magnitude and nature of fault motion. Finally, we suggest that the diffuse plate boundary zone along the CITZ in Central India is a counterpart of the Gakkel Ridge-Chersky Range system in the Arctic, where both display a similar deformation signature due to rotational tectonic deformation that is induced by the impact of an indenter-like structure (Fig. 7). However, we acknowledge that to characterized the complex deformation style along the CITZ in Central India requires more geodetic and seismic station coverage in future.

## Materials and methods

### Source of the GPS sites used in the present study

#### Eurasian Plate

To estimate Eurasian Plate motion and associated crustal deformation covering the entire continental part of the Eurasian Plate and adjacent regions, we have analyzed geodetic data sets from various literatures^10,37–50^. In the present solution, we have considered horizontal site velocities from 684 continuous GPS stations, within the continental part of the Eurasian Plate (Fig. S2), to estimate the absolute and relative rotation parameters to characterize the ongoing seismotectonic activity along the plate boundary of the North American and Eurasian Plates.

#### North American Plate

Similarly, in order to quantify North American Plate motion and associated crustal deformation covering the entire continental part of the North American Plate and adjacent regions, specifically along the plate boundary region shared with Eurasia, we have analyzed geodetic data sets from various previous works^37–40,44,49,51–54^. In the present solution, we have included 2223 continuous GPS velocities from stations within the continental part of North American Plate.

#### Indian Plate

In order to quantify Indian plate motion and associated crustal deformation covering the central continental part of the Indian Plate and adjacent region, we have analyzed geodetic data sets from various published works^29,32,50,54–61^. In the present solution, we have included 28 continuous GPS velocities from stations within the continental Indian plate interior. Further, two blocks Indian ‘composite’ plate has been constrained by 14 GPS sites from the North-Indian Plate and 14 GPS sites from the South-Indian Plate.

### Estimation of Euler rotation parameters

To understand present-day deformation along the plate boundary region between the North American Plate and Eurasian Plate (i.e., Gakkel Ridge to Chersky Range in the Arctic region), and the CITZ, we have estimated Euler rotation parameters (for both absolute and relative Euler rotation poles) of the respective plates or component plates respectively (see Supplementary Materials for more details). Here, we have constrained geodetic cGPS datasets from various open achieve sources, along with longer duration of data and large spatial coverage of large parts of the Indian, North American Plate and Eurasian Plate (see Supplementary Material for more detail). All of these geodetic station velocity fields are subsequently transformed into an uniform and consistent ITRF2014 reference frame (https://www.ngs.noaa.gov/TOOLS/Htdp/Htdp.shtml), in order to minimise the reference frame bias, instead of solving the Helmert transformation parameters as it requires several common sites which are lacking in the present case^62,63^. Here, we have adopted the technique developed by Goudarzi et al.^64^, to estimate the rotation parameters of the respective plates of our interest and the Euler rotation parameters is expressed mathematically as (Tables S1–S5):

1 $${v}_{n}^{p}={\Omega }^{p}\times {X}_{n}={\left[\begin{array}{ccc}0& {-\omega }_{z}& {\omega }_{y}\\ {\omega }_{z}& 0& {-\omega }_{x}\\ {-\omega }_{y}& {\omega }_{x}& 0\end{array}\right]}^{p}{\left[\begin{array}{c}x\\ y\\ z\end{array}\right]}_{n},$$

where ${v}^{p}$ are the velocity and ${\Omega }^{p}\left({\Omega }_{x}^{p}, {\Omega }_{y}^{p}, {\Omega }_{z}^{p}\right)$ is the angular velocity of the Euler rotation pole associated within the plate *p* for the particular station *n.* ${X}_{n} \left({x}_{n}, {y}_{n}, {z}_{n}\right)$ are the position of the cGPS site *n*, which are available in the Local Geodetic Cartesian coordinate system. The station position ${X}_{n}$ is transformed the Local Geodetic Cartesian coordinate system to spherical coordinates, which is expressed mathematically as:

2 $${X}_{n}={R}_{e}{\left[\begin{array}{ccc}0& {\text{sin}}\varphi & -{\text{cos}}\varphi {\text{sin}}\lambda \\ -{\text{sin}}\varphi & 0& {\text{cos}}\varphi {\text{sin}}\lambda \\ {\text{cos}}\varphi {\text{sin}}\lambda & -{\text{cos}}\varphi {\text{sin}}\lambda & 0\end{array}\right]}_{n},$$

where ${R}_{e}$ is the radius of the Earth, $\varphi$ the spherical latitude, and $\lambda$ is the longitude of GPS station *n*^64^.

### Statistical test

In order to present statistical significance in the respective rotation parameters for the Eurasia, North America, and Indian plates respectively (Tables S1–S5), we performed a statistical test Root Mean Square Error (RMSE) analysis. This test evaluates the quality of the velocity datasets for estimation of the Euler rotation parameters. The mathematically RMSE, can be expressed as follows:

3 $${\text{RMSE}}=\sqrt{\frac{\sum_{n=1}^{N}{\left({V}_{on}-{V}_{en}\right)}^{2}}{N},}$$

where, ${V}_{o}$ is the observed velocity, ${V}_{e}$ the expected or predicted velocity, and N the number of data points. Low RMSE values show a statistically more robust dataset.

In addition, we also implement a data snooping approach^65^, which removes the statistically insignificant sites from the analysis or sites with low-reliable data quality. From this analysis, we observe that the statistics of each plates (Eurasia, North American and India Plate) is improved significantly (Tables S1–S5). Similarly, the Euler pole parameters of the absolute independent North Indian plate and South Indian plate becomes more significant after removal of data outliers from the datasets. Also the error (RMSE) of the single Indian Plate analysis is acceptable, though subtle higher than the error (RMSE) of the two respective independent North Indian plate and South Indian plate. Nevertheless, at the time of plate pair pole calculation (Tables S3, S5) for those two regions, we have considered the two component or adjacent plates as the Eurasian and North American Plate in case of the Arctic region and similarly, for the Indian plate, the North Indian plate and South Indian plate has been considered as two component plates. During observation of plate pair pole estimation results for both the cases, it has been noticed that the error (RMSE) value in the statistics tends to increase, possibly indicates the distributed/localized nature of deformation associated in between those component plates.

### Topography analysis

To assess topography changes along the axis of the deformation zone and their relation with the rotational tectonics in both the Gakkel Ridge-Chersky Range system in the Arctic and the CITZ in the Indian province, we generate systematic swath topographic profiles (presented in Figs. 3, 4). These topographic profiles are derived from Global Multi-Resolution Topography (GMRT) and ETOPO1 bathymetry data with a 1-min resolution. Several ~ 50 km-wide topographic swath profiles, perpendicular to the ridge axis across the Arctic region (i.e., from the Gakkel Ridge-Chersky Range system) (Fig. 3b) and the CITZ (Fig. 4b), are produced using the Global Mapper Application.

### Supplementary Information

Supplementary Information.

## Data Availability

Topography profiles are generated using the Global Mapper application, using a topographic database archived from National Oceanic and Atmospheric Administration database (https://maps.ngdc.noaa.gov/viewers/wcs-client/). The Euler pole parameters are estimated using the Euler pole calculator (EPC) archived from National Oceanic and Atmospheric Administration at https://geodesy.noaa.gov/gps-toolbox/Goudarzi.htm. The geodetic station velocity fields are transformed into an uniform and consistent ITRF2014 reference frame (https://www.ngs.noaa.gov/TOOLS/Htdp/Htdp.shtml). All maps and figures are generated by using the Generic Mapping Tools (version 6.3.0; http://gmt.soest.hawaii.edu/). More details on the analogue tectonic models can be found in Zwaan et al.^3^: 10.1016/j.jsg.2019.103946 and in an associated GFZ data publication (Zwaan et al.^66^: 10.5880/fidgeo.2020.001. All other relevant datasets used in this study are mentioned in the text and Supplementary Material and also available at 10.6084/m9.figshare.25225970.v1.
