# Supplementary material for: Unfolding rotational tectonics and topographic evolution from localized verses diffuse plate boundary counterparts
Source: Sci Rep. 2024 Apr 8;14:8199. doi: 10.1038/s41598-024-58921-y (PMC11002002; doi:10.1038/s41598-024-58921-y)
Supplement: Supplementary file 1 — Supplementary Information. [file 41598_2024_58921_MOESM1_ESM.docx]

**Supplementary Material**

**Unfolding rotational tectonics and topographic evolution from localized verses diffuse plate boundary counterparts**

**Bhaskar Kundu^1*^, Frank Zwaan^2,3,4*^ and Batakrushna senapati^1^**

**^1^** Department of Earth and Atmospheric Sciences, NIT Rourkela, Rourkela 769008, India.

**^2^** Institute of Geological Sciences, University of Bern, Baltzerstrasse 1+3, CH-3012, Bern, Switzerland

**^3^** Helmholtz Centre Potsdam - GFZ German Research Centre for Geosciences, Albert-Einstein-Straße 42-46,

14473 Potsdam, Germany

**^4^** University of Fribourg, Department of Geosciences, Ch. du Musée 6, 1700 Fribourg, Switzerland

***Corresponding authors:** Bhaskar Kundu, ([rilbhaskar@gmail.com](mailto:rilbhaskar@gmail.com)) and Frank Zwaan, ([frank.zwaan@gfz-potsdam.de](mailto:frank.zwaan@gfz-potsdam.de))

**Supplementary Figures**


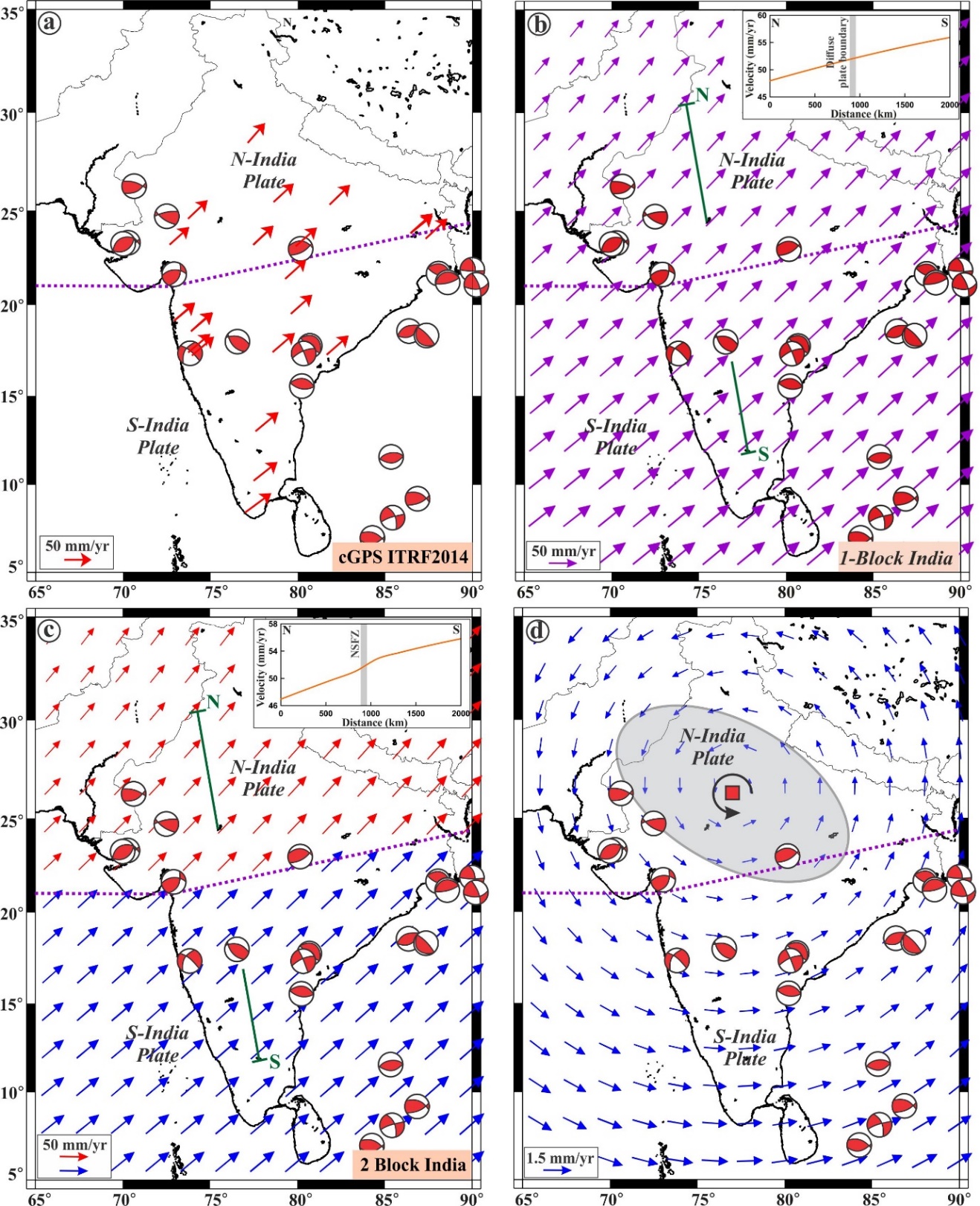


**Fig. S1** (a) Observed cGPS velocity. (b) Predicted velocity by considering Single Indian plate pole. Note that there is no such significant change in velocity across the Narmada-Son diffuse deformation zone in the Inset panel. (c) Predicted velocity by considering Euler rotation poles of the North-Indian (red arrow) and South-Indian plate (blue arrow). Note the South-Indian Plate is moving faster than the North-Indian Plate and hence the existence of deformation along diffuse deformation zone (note the velocity change along Narmada-Son diffuse plate boundary is represented by inset panel). (d) Predicted velocity by considering Geodetic Indian Euler pole. This figure was generated using Generic Mapping Tools (version 5.2.1; URL: [http//gmt.soest.hawaii.edu/](http/gmt.soest.hawaii.edu/)).


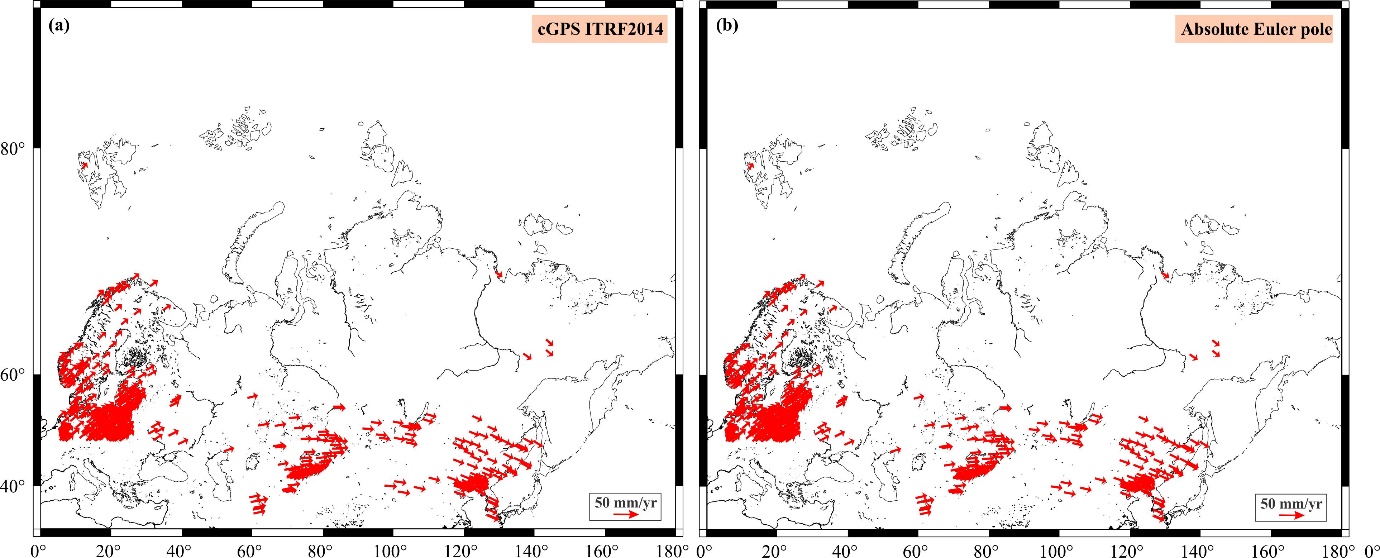


**Fig. S2** (a) Observed cGPS velocity. (b) Predicted velocity by considering Eurasia pole. This figure was generated using Generic Mapping Tools (version 5.2.1; URL: [http//gmt.soest.hawaii.edu/](http/gmt.soest.hawaii.edu/)).

**Supplementary Tables**

**Table S1.** Eurasia Plate Euler Pole Parameters proposed by previous (black) and present study (in bold).

| **Sr. No** | **Latitude (°N)** | **Longitude (°E)** | **Omega (°/Myr)** | **No of Sites Used** | **Model type** | **Reference** |
| --- | --- | --- | --- | --- | --- | --- |
| (1) | 61.066 | -85.819 | 0.819 | _ | NUVEL-1A | Demets et al^1^ |
| (2) | 56.3±5.7 | -102.8±1.7 | 0.26±0.02 | 8 | ITRF1994 | Larson et al^2^ |
| (3) | 58.27±1.5 | -102.21±0.4 | 0.257±0.003 | 15 | ITRF1997 | Sella et al.^3^ |
| (4) | 57.965±1.211 | -99.374±2.710 | 0.260±0.005 | 20 | ITRF2000 | Altamimi et al^4^ |
| (5) | 58.3±1.5 | -97.2±0.3 | 0.260±0.001 | 18 | ITRF2000 | Bock et al^5^ |
| (6) | 54.6±1.6 | -103.9±0.4 | 0.249±0.003 | 58 | ITRF2000 | Fernandes et al^6^ |
| (7) | 52.3±0.2 | -107.0±0.2 | 0.245±0.005 | 15 | ITRF2000 | Calais et al^7^ |
| (8) | 56.4±0.6 | -97.4±0.2 | 0.27±0.005 | _ | NNR | Kreemer et al^8^ |
| (9) | 57.2±0.8 | -99.7±0.2 | 0.260±0.002 | 18 | ITRF2000 | Prawirodirdjo et al^9^ |
| (10) | 55.6±1.8 | -102.4±0.9 | 0.252±0.010 | 11 | ITRF2000 | Shen et al^10^ |
| (11) | 56.33±0.0 | -95.979±0.2 | 0.261±0.003 | 41 | ITRF2005 | Altamimi et al^11^ |
| (12) | 24.38± 0.42 | 102.37± 0.42 | 0.7096 ±0.0206 | _ | _ | Gan et al^12^ |
| (13) | 55.339±0.0 | -105.022±0.0 | 0.260±0.001 | 19 | ITRF2000 | Apel^13^ |
| (14) | 55.47 | -99.88 | 0.254 | 8 | ITRF2008 | Kreemer et al^14^ |
| (15) | 54.20 | -99.95 | 0.258 | 60 | ITRF2014 | Demets et al^15^ |
| (16) | **55.544±0.103** | **-97.589±0.138** | **0.254±0.0004** | **566** | **ITRF2014** | **Present Study** |

**Table S2.** North America Plate Euler Pole Parameters proposed by previous (black) and present study (in bold).

| **Sr.**  **No** | **Latitude (°N)** | **Longitude (°E)** | **Omega (°/Myr)** | **No of Sites Used** | **Model type** | **Reference** |
| --- | --- | --- | --- | --- | --- | --- |
| (1) | 48.709 | -78.167 | 0.7486 | _ | NUVEL-1A | DeMets et al^1^ |
| (2) | -0.4±4.3 | -84.5±2.0 | 0.22±0.1 | 9 | ITRF1994 | Larson et al^2^ |
| (3) | -0.9±4.1 | -79.8±1.6 | 0.192±0.009 | 16 | ITRF1996 | DeMets and Dixon^16^ |
| (4) | -1.88±1.04 | -77.67±0.39 | 0.201±0.004 | 55 | ITRF1997 | Gan et al^17^ |
| (5) | -2.39 | -79.08 | 0.199 | 64 | ITRF1997 | Sella et al^3^ |
| (6) | -3.86 | -83.96 | 0.199±0.003 | _ | ITRF2000 | Beavan et al^18^ |
| (7) | -5.036±1.142 | -83.144±1.945 | 0.194±0.003 | 16 | ITRF2000 | Altamimi et al^4^ |
| (8) | 50.8 | -77.9 | 0.768 | _ | NUVEL-1A | Kreemer et al^8^ |
| (9) | 1*.*7 | -82*.*3 | 0*.*211 | _ | NNR | Kreemer et al^8^ |
| (10) | 6*.*8 | -95*.*2 | 0.189 | 457 | MORVEL | DeMets et al^19^ |
| (11) | -1.40 | -86.22 | 0.207 | 10 | ITRF2008 | Kreemer et al^14^ |
| (12) | **-7.926±0.115** | **-87.797±0.030** | **0.181±0.0003** | **2103** | **ITRF2014** | **Present Study** |

**Table S3.** Plate Pair or relative Euler Pole Parameters between Eurasia and North America proposed by previous (black) and present study (in bold).

| **Sr. No** | **Latitude (°N)** | **Longitude (°E)** | **Omega**  **(°/Myr)** | **No of Sites Used** | **Model type** | **Reference** |
| --- | --- | --- | --- | --- | --- | --- |
| (1) | 62.4±4.1 | 135.8±1.3 | 0.21±0.01 | 39 | NUVEL-1A | Demets et al^1^ |
| (2) | 78.5±8.2 | 122.0±4.9 | 0.23±0.03 | _ | JPL-GPS | Argus and Hefin^20^ |
| (3) | 66.7±3.0 | 126.8±1.2 | 0.22±0.01 | _ | Goddard | Smith et al^21^ |
| (4) | 68.1±6.5 | 126.6±3.9 | 0.24±0.02 | _ | ITRF1994 | Larson et al^2^ |
| (5) | 74.0±5.4 | 111.3±2.4 | 0.26±0.02 | _ | VLBI | Argus and Gordon^22^ |
| (6) | 74.30±1.8 | 123.0±1.4 | 0.231±0.008 | _ | GPS/VLBI | Kogan et al^23^ |
| (7) | 68.05±1.5 | 136.42±0.8 | 0.245±0.004 | _ | REVEL-2000 | Sella et al^3^ |
| (8) | 73.032 | 128.99 | 0.248 | _ | ITRF2000 | Altamimi et al^4^ |
| (9) | **75.40±1.7** | **124.2±1.5** | **0.233±0.008** | **_** | **ITRF2014** | **Present Study** |

**Table S4.** Geodetic Indian Euler Plate rotation parameters proposed by previous (black) and present study (in bold).

| **Sr.**  **No** | **Latitude (°N)** | **Longitude (°E)** | **Omega (°/Myr)** | **No of Sites Used** | **Model type** | **Reference** |
| --- | --- | --- | --- | --- | --- | --- |
| (1) | 53.7±11.7 | −13.9±0.5 | 0.483 ±0.013 | 3 | ITRF1997 | Sella et al^3^ |
| (2) | 53.1 | 2.2 | 0.519±0.019 | – | ITRF2000 | SOPAC Website^24^ |
| (3) | 45.72±12.1 | -41.99±0.73 | 0.487±0.015 | 2 | ITRF2000 | Prawirodirdjo et al^9^ |
| (4) | 51.4±1.6 | −10.9±5.6 | 0.483±0.01 | 5 | ITRF2000 | Bettinelli et al^25^ |
| (5) | 50.9±5.1 | −12.1±0.6 | 0.486±0.001 | 6 | ITRF2000 | Socquet et al^26^ |
| (6) | 51.7±0.5 | −15.1±1.5 | 0.469±0.01 | 26* | ITRF2000 | Jade et al^27^ |
| (7) | 52.9±0.21 | −0.297±3.760 | 0.499±0.008 | 12 | ITRF2000 | Banerjee et al^28^ |
| (8) | 51.4±0.3 | -1.34±3.31 | 0.503±0.007 | 20 | ITRF2005 | Ader et al^29^ |
| (9) | 51.4±0.07 | 8.9±0.8 | 0.539±0.002 | 13 | ITRF2008 | Mahesh et al^30^ |
| (10) | 51.42±0.06 | 2.10±0.38 | 0.5146±0.001 | 15 | ITRF2008 | Steckler et al^31^ |
| (11) | 51.69±0.271 | 11.85±1.79 | 0.553±0.005 | 30* | ITRF2008 | Jade et al^32^ |
| (12) | 51.51 | -4.00 | 0.509 | 29 | _ | Demets et al^15^ |
| (13) | **51.33±0.181** | **9.45±1.991** | **0.541±0.006** | **24** | **ITRF2014** | **Present Study (1-Plate)** |
| (14) | **51.48±0.335**  **51.55±0.146** | **13.68±4.637**  **4.68±1.084** | **0.553±0.019**  **0.528±0.002** | **12**  **13** | **ITRF2014** | **Present Study (2-Plate)**  **N-India**  **S-India** |

*Sites considered by Jade et al^27^ (2007) and Jade et al^32^ to characterize the Euler rotation parameter of the Indian plate. Although the number of sites are more than the present study but the geodetic sites considered in Jade et al^27^ and Jade et al^32^ are mainly affected by post seismic and internal deformation process.

**Table S5.** Plate Pair or relative Euler Pole Parameters between North-India and South-India

| **Sr. No** | **Latitude (°N)** | **Longitude (°E)** | **Omega (°/Myr)** | **No of Sites used** | **Model type** | **Reference** |
| --- | --- | --- | --- | --- | --- | --- |
| **(1)** | **25.92±6.806** | **77.09±4.09** | **0.035±0.021** | **28** | **ITRF2014** | **Present Study**  **Composite Euler Pole (N-India/S-India)** |
| **(2)** | **22.14** | **72.92** | **0.162** | – | – | **Apel**^13^ |

**Table S6.** Eurasia Plate Euler Pole parameters proposed by previous (black) and present study (in bold) and RMSE values between GNSS velocities and kinematic velocities

| **Latitude (°N)** | **Longitude (°E)** | **Omega (°/Myr)** | **Reference** | **RMSE** |
| --- | --- | --- | --- | --- |
| 61.066 | -85.819 | 0.819 | Demets et al^1^ | 45.79 |
| 56.3±5.7 | -102.8±1.7 | 0.26±0.02 | Larson et al^2^ | 7.01 |
| 58.27±1.5 | -102.21±0.4 | 0.257±0.003 | Sella et al.^3^ | 6.75 |
| 58.3±1.5 | -97.2±0.3 | 0.260±0.001 | Bock et al^5^ | 6.80 |
| 54.6±1.6 | -103.9±0.4 | 0.249±0.003 | Fernandes et al^6^ | 6.62 |
| 52.3±0.2 | -107.0±0.2 | 0.245±0.005 | Calais et al^7^ | 6.72 |
| 56.4±0.6 | -97.4±0.2 | 0.27±0.005 | Kreemer et al^8^ | 7.43 |
| 57.2±0.8 | -99.7±0.2 | 0.260±0.002 | Prawirodirdjo et al^9^ | 6.90 |
| 55.6±1.8 | -102.4±0.9 | 0.252±0.010 | Shen et al^10^ | 6.55 |
| 56.33±0.0 | -95.979±0.2 | 0.261±0.003 | Altamimi et al^11^ | 6.88 |
| 55.339±0.0 | -105.022±0.0 | 0.260±0.001 | Apel^13^ | 7.11 |
| 55.47 | -99.88 | 0.254 | Kreemer et al^14^ | 6.83 |
| 54.20 | -99.95 | 0.258 | Demets et al^15^ | 6.86 |
| **55.544±0.103** | **-97.589±0.138** | **0.254±0.0004** | **Present Study** | **6.54** |

**Table S7.** North America Plate Euler Pole parameters proposed by previous (red) and present study (in bold) and RMSE values between GNSS velocities and kinematic velocities

| **Latitude (°N)** | **Longitude (°E)** | **Omega (°/Myr)** | **Reference** | **RMSE** |
| --- | --- | --- | --- | --- |
| -0.4±4.3 | -84.5±2.0 | 0.22±0.1 | Larson et al^2^ | 5.85 |
| -1.88±1.04 | -77.67±0.39 | 0.201±0.004 | Gan et al^17^ | 5.47 |
| -2.39 | -79.08 | 0.199 | Sella et al^3^ | 5.38 |
| -3.86 | -83.96 | 0.199±0.003 | Beavan et al^18^ | 5.50 |
| -5.036±1.142 | -83.144±1.945 | 0.194±0.003 | Altamimi et al^4^ | 5.45 |
| 50.8 | -77.9 | 0.768 | Kreemer et al^8^ | 5.45 |
| 6*.*8 | -95*.*2 | 0.189 | DeMets et al^19^ | 8.54 |
| -1.40 | -86.22 | 0.207 | Kreemer et al^14^ | 10.79 |
| **-7.926±0.115** | **-87.797±0.030** | **0.181±0.0003** | **Present Study** | **5.35** |

**Table S8.** Geodetic Indian Euler Plate rotation parameters proposed by previous (black) and present study (in bold) and RMSE values between GNSS velocities and kinematic velocities

| **Latitude (°N)** | **Longitude (°E)** | **Omega (°/Myr)** | **Reference** | **RMSE** |
| --- | --- | --- | --- | --- |
| 53.7±11.7 | −13.9±0.5 | 0.483 ±0.013 | Sella et al^3^ | 4.37 |
| 53.1 | 2.2 | 0.519±0.019 | SOPAC Website^24^ | 4.06 |
| 45.72±12.1 | -41.99±0.73 | 0.487±0.015 | Prawirodirdjo et al^9^ | 5.77 |
| 50.9±5.1 | −12.1±0.6 | 0.486±0.001 | Socquet et al^26^ | 3.71 |
| 51.7±0.5 | −15.1±1.5 | 0.469±0.01 | Jade et al^27^ | 3.93 |
| 52.9±0.21 | −0.297±3.760 | 0.499±0.008 | Banerjee et al^28^ | 3.54 |
| 51.4±0.3 | -1.34±3.31 | 0.503±0.007 | Ader et al^29^ | 3.81 |
| 51.4±0.07 | 8.9±0.8 | 0.539±0.002 | Mahesh et al^30^ | 3.59 |
| 51.42±0.06 | 2.10±0.38 | 0.5146±0.001 | Steckler et al^31^ | 3.53 |
| 51.51 | -4.00 | 0.509 | Demets et al^15^ | 3.67 |
| **51.33±0.181** | **9.45±1.991** | **0.541±0.006** | **Present Study (1-Plate)** | **3.52** |
| **51.48±0.335**  **51.55±0.146** | **13.68±4.637**  **4.68±1.084** | **0.553±0.019**  **0.528±0.002** | **Present Study (2-Plate)**  **N-India**  **S-India** | **3.51**  **2.31** |

**Table S9.** Analogue modelling related model properties, model characteristics and scaling parameters^33^.

| **Material**  **properties** | **Granular materials** | **Quartz sand^a^** | **Corundum sand^b^** |  |
| --- | --- | --- | --- | --- |
|  | Grain size range (ø) | 60–250 μm | 88–125 μm |  |
|  | Specific density^c^ (ρspecific) | 2650 kg/m3 | 3950 kg/m3 |  |
|  | Sieved density (ρsieved) | 1560 kg/m3 | 1890 kg/m3 |  |
|  | Angle of internal peak friction | 36.1° | 37° |  |
|  | Angle of dynamic-stable friction | 31.4° | 32° |  |
|  | Cohesion | 9 ± 98 Pa | 39 ± 10 Pa |  |
|  | **Viscous materials** | **Pure PDMS^d^** | **PDMS/corundum sand mixture^a^** |  |
|  | Weight ratio PDMS: corundum sand | __ | 0.965 kg: 1.00 kg |  |
|  | Density (ρ) | 965 kg/m^3^ | ca. 1600 kg/m^3^ |  |
|  | Viscosity (η) | ca. 2.5∙104 Pa∙s | ca. 1.5∙105 Pa∙s^e^ |  |
|  | Type^f^ | Newtonian (n = ca. 1) | near-Newtonian (n=1.05–1.10) |  |
| **Model**  **characteristics** | **Model** | **Seed length (L)** | **CT- scanned** | **Shown in** |
|  | Rotational extension | Full (65 + 25 cm) | yes | Fig. 5 |
| **Scaling parameters** |  | **Model** | **Nature (continent)** | **Nature (ocean)** |
| General parameters | Gravitational accel. (g) | 9.81 m/s^2^ | 9.81 m/s^2^ | 9.81 m/s^2^ |
|  | Extension velocity (v) | 2.2∙10^-6^ m/s | 1.5∙10^-10^ m/s | 3.6∙10^-10^ m/s |
| Brittle layer | Material/represents | Sand layer | Upper crust | Oceanic crust |
|  | Thickness (h) | 4∙10^-2^ | 2∙10^4^ | 7∙10^3^ |
|  | Density (ρ) | 1560 kg/m^3^ | 2800 kg/m^3^ | 3000 kg/m^3^ |
|  | Cohesion (C) | 9 Pa | 8∙10^6^ Pa | 3∙10^6^ Pa |
| Viscous/ductile layer | Material/represents | PDMS/cor. sand mix | Lower crust | Lithospheric mantle |
|  | Thickness (h) | 4∙10^-2^ m | 2∙10^4^ m | 4∙10^-2^ m |
|  | Density (ρ) | 1600 kg/m^3^ | 2870 kg/m^3^ | 3075 kg/m^3^ |
|  | Viscosity (η) | 1.5∙10^5^ Pa∙s | 1∙10^21^ Pa∙s | 5∙10^19^ Pa.s |
| Dynamic scaling values | Brittle stress ratio (R_s_) | 68 | 68 | 69 |
|  | Ramberg number (R_m_) | 75 | 75 | 81 |

^a^ Quartz sand and viscous mixture characteristics after Zwaan et al^34-35^.

^b^ Corundum sand characteristics after Panien et al^36^

^c^ Specific densities after Carlo AG ^37^.

^d^ Pure PDMS rheology after Rudolf et al^38^

^e^ Viscosity value holds for model strain rates *<* 10^-4^ S^-1^.

^f^ Power-law exponent n (dimensionless) represents sensitivity to strain rate.

**Supporting References:**

1. DeMets, C., Gordon, R.G., Argus, D.F. and Stein, S. Effect of recent revisions to the geomagnetic reversal time scale on estimates of current plate motions. *Geophys. Res. Lett.* **21**(20), 2191-2194 (1994). <https://doi.org/10.1029/94GL02118>
2. Larson, K.M., Freymueller, J.T. and Philipsen, S., Global plate velocities from the Global Positioning System. *J. Geophys. Res. Solid Earth.* **102**(B5), 9961-9981 (1997). <https://doi.org/10.1029/97JB00514>
3. Sella, G.F., Dixon, T.H. and Mao, A. REVEL: A model for recent plate velocities from space geodesy. *J. Geophys. Res. Solid Earth.* **107**(B4), ETG 11-1-ETG 11-30 (2002). <https://doi.org/10.1029/2000JB000033>
4. Altamimi, Z., Sillard, P. and Boucher, C. ITRF2000: A new release of the International Terrestrial Reference Frame for earth science applications. *J. Geophys. Res. Solid Earth.* **107**(B10), ETG-2 (2002). <https://doi.org/10.1029/2001JB000561>
5. Bock, Y.E.H.U.D.A., Prawirodirdjo, L., Genrich, J.F., Stevens, C.W., McCaffrey, R., Subarya, C., Puntodewo, S.S.O. and Calais, E., 2003. Crustal motion in Indonesia from global positioning system measurements. *J. Geophys. Res. Solid Earth.* **108**(B8) (2003). <https://doi.org/10.1029/2001JB000324>
6. Fernandes, R.M.S., Ambrosius, B.A.C., Noomen, R., Bastos, L., Wortel, M.J.R., Spakman, W. and Govers, R. The relative motion between Africa and Eurasia as derived from ITRF2000 and GPS data. *Geophys. Res. Lett.* **30**(16) (2003). <https://doi.org/10.1029/2003GL017089>
7. Calais, E., Vergnolle, M., San'Kov, V., Lukhnev, A., Miroshnitchenko, A., Amarjargal, S. and Déverchère, J. GPS measurements of crustal deformation in the Baikal‐Mongolia area (1994–2002): Implications for current kinematics of Asia. *J. Geophys. Res. Solid Earth.* **108**(B10) (2003). <https://doi.org/10.1029/2002JB002373>
8. Kreemer, C., Holt, W.E. and Haines, A.J. An integrated global model of present-day plate motions and plate boundary deformation. *Geophys. J. Int.* **154**(1), 8-34 (2003). <https://doi.org/10.1046/j.1365-246X.2003.01917.x>
9. Prawirodirdjo, L. and Bock, Y. Instantaneous global plate motion model from 12 years of continuous GPS observations. *J. Geophys. Res. Solid Earth.* **109**(B8) (2004). <https://doi.org/10.1029/2003JB002944>
10. Shen, Z.K., Lü, J., Wang, M. and Bürgmann, R. Contemporary crustal deformation around the southeast borderland of the Tibetan Plateau. *J. Geophys. Res. Solid Earth.* **110**(B11) (2005). <https://doi.org/10.1029/2004JB003421>
11. Altamimi, Z., Collilieux, X., Legrand, J., Garayt, B. and Boucher, C. ITRF2005: A new release of the International Terrestrial Reference Frame based on time series of station positions and Earth Orientation Parameters. *J. Geophys. Res. Solid Earth.* **112**(B9) (2007). <https://doi.org/10.1029/2007JB004949>
12. Gan, W., Zhang, P., Shen, Z.K., Niu, Z., Wang, M., Wan, Y., Zhou, D. and Cheng, J. Present‐day crustal motion within the Tibetan Plateau inferred from GPS measurements. *J. Geophys. Res. Solid Earth.* **112**(B8) (2007). <https://doi.org/10.1029/2005JB004120>
13. Apel, E. V. Shells on a Sphere: Tectonic Plate Motion and Plate Boundary Deformation. *UC Berkeley. ProQuest ID: Apel_berkeley_0028E_11722. Merritt ID: ark:/13030/m5jm2fmr* (2011)*.* Retrieved from <https://escholarship.org/uc/item/7xm5z7bb>
14. Kreemer, C., Blewitt, G., & Klein, E. C. A geodetic plate motion and Global Strain Rate Model. Geochem Geophys. **15**(10), 3849-3889 (2014). <https://doi.org/10.1002/2014GC005407>
15. DeMets, C., Merkouriev, S. and Jade, S. High-resolution reconstructions and GPS estimates of India–Eurasia and India–Somalia plate motions: 20 Ma to the present. *Geophys. J. Int.* **220**(2), 1149-1171 (2020). <https://doi.org/10.1093/gji/ggz508>
16. DeMets, C. and Dixon, T.H. New kinematic models for Pacific‐North America motion from 3 Ma to present, I: Evidence for steady motion and biases in the NUVEL‐1A model. *Geophys. Res. Lett.* **26**(13), 1921-1924 (1999). <https://doi.org/10.1029/1999GL900405>
17. Gan, W. and Prescott, W.H. Crustal deformation rates in central and eastern US inferred from GPS. *Geophys. Res. Lett.* **28**(19), 3733-3736 (2001). <https://doi.org/10.1029/2001GL013266>
18. Beavan, J., Tregoning, P., Bevis, M., Kato, T. and Meertens, C. Motion and rigidity of the Pacific Plate and implications for plate boundary deformation. *J. Geophys. Res. Solid Earth.* **107**(B10), ETG-19 (2002). <https://doi.org/10.1029/2001JB000282>
19. DeMets, C., Gordon, R.G. and Argus, D.F. Geologically current plate motions. *Geophys. J. Int.*  181(1),1-80 (2010). <https://doi.org/10.1111/j.1365-246X.2009.04491.x>
20. Argus, D.F. and Heflin, M.B. Plate motion and crustal deformation estimated with geodetic data from the Global Positioning System. *Geophys. Res. Lett.* **22**(15), 1973-1976 (1995). <https://doi.org/10.1029/95GL02006>
21. Smith, D.E., Kolenkiewicz, R., Robbins, J.W., Torrence, M.H., Heflin, M. and Soudarin, L. A space geodetic plate motion model. *Eos Transactions, American Geophysical Union*, *77*, p. S73 (1996).
22. Argus, D.F. and Gordon, R.G. Tests of the rigid-plate hypothesis and bounds on intraplate deformation using geodetic data from very long baseline interferometry. *J. geophys. Res.* **101**, 13 555–13 572 (1996). <https://doi.org/10.1029/95JB03775>
23. Kogan, M.G., Steblov, G.M., King, R.W., Herring, T.A., Frolov, D.I., Egorov, S.G., Ye. Levin, V., Lerner‐Lam, A. and Jones, A. Geodetic constraints on the rigidity and relative motion of Eurasia and North America. *Geophys. Res. Lett.* **27**(14), 2041-2044 (2000). <https://doi.org/10.1029/2000GL011422>
24. Scripps Orbit and Permanent Array Center (SOPAC) Website: <http://sopaccsrc.ucsd.edu/index.php/sopac/>
25. Bettinelli, P., Avouac, J.P., Flouzat, M., Bollinger, L., Ramillien, G., Rajaure, S. and Sapkota, S., 2008. Seasonal variations of seismicity and geodetic strain in the Himalaya induced by surface hydrology. *Earth Planet. Sci. Lett.* **266**(3-4), 332-344 (2008). <https://doi.org/10.1016/j.epsl.2007.11.021>
26. Socquet, A., Vigny, C., Chamot‐Rooke, N., Simons, W., Rangin, C. and Ambrosius, B. India and Sunda plates motion and deformation along their boundary in Myanmar determined by GPS. *J. Geophys. Res. Solid Earth.* **111**(B5) (2006). <https://doi.org/10.1029/2005JB003877>
27. Jade, S., Mukul, M., Bhattacharyya, A.K., Vijayan, M.S.M., Jaganathan, S., Kumar, A., Tiwari, R.P., Kumar, A., Kalita, S., Sahu, S.C. and Krishna, A.P., 2007. Estimates of interseismic deformation in Northeast India from GPS measurements. *Earth Planet. Sci. Lett.* **263**(3-4), 221-234 (2007). <https://doi.org/10.1016/j.epsl.2007.08.031>
28. Banerjee, P., Bürgmann, R., Nagarajan, B. and Apel, E. Intraplate deformation of the Indian subcontinent. *Geophys. Res. Lett.* **35**(18) (2008). <https://doi.org/10.1029/2008GL035468>
29. Ader, T., Avouac, J.P., Liu‐Zeng, J., Lyon‐Caen, H., Bollinger, L., Galetzka, J., Genrich, J., Thomas, M., Chanard, K., Sapkota, S.N. and Rajaure, S. Convergence rate across the Nepal Himalaya and interseismic coupling on the Main Himalayan Thrust: Implications for seismic hazard. *J. Geophys. Res. Solid Earth.* **117**(B4) (2012). <https://doi.org/10.1029/2011JB009071>
30. Mahesh, P., Catherine, J.K., Gahalaut, V.K., Kundu, B., Ambikapathy, A., Bansal, A., Premkishore, L., Narsaiah, M., Ghavri, S., Chadha, R.K. and Choudhary, P. Rigid Indian plate: constraints from GPS measurements. *Gondwana Research*, **22**(3-4), 1068-1072 (2012). <https://doi.org/10.1016/j.gr.2012.01.011>
31. Steckler, M.S., Mondal, D.R., Akhter, S.H., Seeber, L., Feng, L., Gale, J., Hill, E.M. and Howe, M. Locked and loading megathrust linked to active subduction beneath the Indo-Burman Ranges. *Nat. Geosci.* **9**(8), 615-618 (2016). <https://doi.org/10.1038/ngeo2760>
32. Jade, S., Shrungeshwara, T.S., Kumar, K., Choudhury, P., Dumka, R.K. and Bhu, H. India plate angular velocity and contemporary deformation rates from continuous GPS measurements from 1996 to 2015. *Sci. Rep*. **7**(1), 1-16 (2017). <https://doi.org/10.1038/s41598-017-11697-w>
33. Zwaan, F., Schreurs, G. and Rosenau, M. 2020. Rift propagation in rotational versus orthogonal extension: Insights from 4D analogue models. *J. Struct. Geol.* ***135***, 103946 (2020). <https://doi.org/10.1016/j.jsg.2019.103946>
34. Zwaan, F., Schreurs, G., Naliboff, J. and Buiter, S.J. Insights into the effects of oblique extension on continental rift interaction from 3D analogue and numerical models. *Tectonophysics*, **693**, 239-260 (2016). <https://doi.org/10.1016/j.tecto.2016.02.036>
35. Zwaan, F., Schreurs, G., Gentzmann, R., Warsitzka, M. and Rosenau, M. Ring-shear test data of quartz sand from the Tectonic Modelling Lab of the University of Bern (CH) (2018). <https://doi.org/10.5880/fidgeo.2018.028>
36. Panien, M., Schreurs, G., Pfiffner, A. Mechanical behaviour of granular materials used in analogue modelling: insights from grain characterisation, ring-shear tests and analogue experiments. *J. Struct. Geol.* **28**, 1710–1724 (2006). https://doi.org/10.1016/ j.jsg.2006.05.004
37. Carlo AG (Carlo Bernasconi AG, Switzerland), (2019). Company website. [www.carloag.ch](http://www.carloag.ch)
38. Rudolf, M., Boutelier, D., Rosenau, M., Schreurs, G., Oncken, O. Rheological benchmark of silicone oils used for analog modeling of short- and long-term lithospheric deformation. *Tectonophysics,* **684**, 12–22 (2016). [https://doi.org/10.1016/j. tecto.2015.11.028](https://doi.org/10.1016/j.%20tecto.2015.11.028)
